# Aroma Profile and Biological Effects of *Ochradenus arabicus* Essential Oils: A Comparative Study of Stem, Flowers, and Leaves

**DOI:** 10.3390/molecules27165197

**Published:** 2022-08-15

**Authors:** Obaid Ullah, Muddaser Shah, Najeeb Ur Rehman, Saeed Ullah, Jamal Nasser Al-Sabahi, Tanveer Alam, Ajmal Khan, Nasir Ali Khan, Naseem Rafiq, Saqib Bilal, Ahmed Al-Harrasi

**Affiliations:** 1 Natural and Medical Sciences Research Center, University of Nizwa, Birkat Al Mauz, P.O. Box 33, Nizwa 616, Oman; 2 Department of Chemistry, University of Malakand, Chakdara Dir Lower 18800, Pakistan; 3 Department of Botany, Abdul Wali Khan University Mardan, Mardan 23200, Pakistan; 4 Central Instrumentation Laboratory, Medical Research Center, College of Agricultural and Marine Sciences, Sultan Qaboos University, Muscat 123, Oman; 5 Department of Zoology, Abdul Wali Khan University Mardan, Mardan 23200, Pakistan

**Keywords:** *Ochradenus arabicus*, GC-MS analysis, essential oils, antimicrobial, antidiabetics, antioxidant

## Abstract

The present analysis explores the chemical constituents and determines the in vitro antimicrobial, antidiabetic, and antioxidant significance of the essential oils (EOs) of the stem, leaves, and flowers of *Ochradenus arabicus* for the first time. The EOs of the flowers presented seventy-four constituents contributing to 81.46% of the total EOs, with the major compounds being 24-norursa-3,12-diene (13.06%), 24-norursa-3,12-dien-11-one (6.61%), and 24-noroleana-3,12-diene (6.25%). The stem EOs with sixty-one compounds contributed 95.95% of the total oil, whose main bioactive compounds were (+)-camphene (21.50%), eremophilene (5.87%), and δ-selinene (5.03%), while a minimum of fifty-one compounds in the leaves’ EOs (98.75%) were found, with the main constituents being *n*-hexadecanoic acid (12.32%), octacosane (8.62%), tetradecanoic acid (8.54%), and prehydro fersenyl acetone (7.27%). The antimicrobial activity of the EOs of *O. arabicus* stem, leaves, and flowers was assessed against two bacterial strains (*Escherichia* *coli* and *Streptococcus aureus*) and two fungal strains (*Penicillium simplicissimum* and *Rhizoctonia* *solani*) via the disc diffusion assay. However, the EOs extracted from the stem were found effective against one bacterial strain, *E. coli*, and one fungal strain, *R. Solani*, among the examined microbes in comparison to the standard and negative control. The tested EOs samples of the *O. arabicus* stem displayed a maximum potential to cure diabetes with an IC_50_ = 0.40 ± 0.10 µg/mL, followed by leaves and flowers with an IC_50_ = 0.71 ± 0.11 µg/mL and IC_50_ = 10.57 ± 0.18 µg/mL, respectively, as compared to the standard acarbose (IC_50_ = 377.26 ± 1.20 µg/mL). In addition, the EOs of *O. arabicus* flowers had the highest antioxidant activity (IC_50_ = 106.40 ± 0.19 µg/mL) as compared to the standard ascorbic acid (IC_50_ = 73.20 ± 0.17 µg/mL) using the 2,2-diphenyl-1-picrylhydrazyl (DPPH) assay. In the ABTS assay, the EOs of the same sample (flower) depicted the utmost potential to scavenge the free radicals with an IC_50_ = 178.0 ± 0.14 µg/mL as compared with the ascorbic acid, having an IC_50_ of 87.34 ± 0.10 µg/mL the using 2,2-Azino-Bis-3-Ethylbenzothiazoline-6-Sulfonic acid (ABTS) assay. The EOs of all parts of *O. arabicus* have useful bioactive components due to which they present antidiabetic and antioxidant significance. Furthermore, additional investigations are considered necessary to expose the responsible components of the examined biological capabilities, which would be effective in the production of innovative drugs.

## 1. Introduction

The genus *Ochradenus* belongs to the family Resedaceae, is represented by eight species, and is mostly distributed in the desert and arid regions of the Arabian Peninsula, Southwest Asia, and Northeast Africa [1]. Only one species (*O. baccatus* Del.) of the genus is widely distributed across the world, while the rest of the species is endemic to the Arabian Peninsula, especially Oman, and Africa [2,3]. An aqueous alcohol extract of *O. baccatus*, in a survey conducted on Egyptian desert plants, was shown to reduce blood cholesterol levels in rats [4]. In addition, the aqueous extract from the aerial parts of *O. baccatus* possesses an antitumor effect against human liver cancer cells [4]. The genus *Ochradenus* has diverse biomedical applications and is reported for its antidiabetic, antimicrobial, hepatoprotective, anticancer, and antioxidant significance [5,6,7], which may be due to the presence of previously reported bioactive compounds, such as flavonoids, flavonoid glycosides, and triterpenoids [8].

*Ochradenus arabicus* Chaudhary, Hillc. & A. G. Mill. is a perennial shrub that grows in the desert, sandy, and arid regions of Oman, United Arab Emirates, Saudi Arabia, and Yemen [9,10]. Previous reports determined that the ethyl acetate fraction of *O. arabicus* was found to be significantly rich in antioxidants, total phenolics, and total flavonoids [5]. Furthermore, Ali et al. [8] reported the cytotoxic potential and chromatographic profiling of a crude ethanolic extract of *O. arabicus* [8,11]. This plant has been reported for its effective propagation and conservation through tissues culture techniques [9,12] as well as different biological activities, including antibacterial, anti-malarial, anti-inflammatory, and, even, anti-cancer, antimicrobial, antidiabetic, phenolics, allopathic, antioxidant, flavonoids, and nutritional properties [4,5,13]. Recently, Shaikhaldein et al. [14] synthesized silver nanoparticles (AgNPs) of *O. arabicus* leaves extract and investigated their effect on the morphophysiological properties of *Maerua oblongifolia* raised in vitro. The ameliorative effect of zinc oxide nanoparticles (ZnONPs) derived from *O. arabicus* leaf extract by green technology against PB-induced toxicity in Swiss albino rats was reported by Hassan et al. [15]. A previous phytochemical investigation of *O. arabicus* resulted in the isolation of cyclopropyl-triterpenoid, octacosan-1-ol, pentacosanoic acid (3), β-sitosterol (4), and β-sitosterol 3-*O*-β-d-glucopyranoside (Hussain et al. [5]). However, there is no report available in the literature on the essential oil composition of this plant. 

Diabetes mellitus (DM) is the most prevalent complication and is associated with insulin deficiency and may lead to a risk of other incurable disorders, such as cancer, inflammation, pain, and cardiovascular complications [16]. DM comprises numerous types, including insulin-deficient type 2 diabetes mellitus (T2DM), which will account for around 90% of cases and may affect approximately 300 million people around the globe by 2025 [17]. T2DM is a frequent metabolic syndrome, characterized by a high level of glucose and responsible for hyperglycemia, which affects the insulin balance and further leads to excessive hunger, thirst, and abnormal urination [18]. Clinically approved inhibitors, such as acarbose, voglibose, and miglitol, are commercially distributed antidiabetic drugs to reduce blood glucose levels for a short period but have adverse effects, such as diarrhea, flatulence, and abdominal disorders [19]. Plant essential oils have an alternative role in coping with diabetes, which is less toxic and much more effective. The α-Glucosidase enzyme has been considered a crucial and key target for curing diabetes, and the inhibitors of this enzyme can be used as effective therapeutic medications to treat diabetes [20]. Therefore, to overcome the side effects of these drugs, there is an urgent need for medicinal chemists to discover effective and safe α-glucosidase inhibitors. The increase in diseases caused by the microbes and adverse effects of the available commercial drugs, influence scientists to search for medicinal plants and their yields, among which the EOs offer rapid consideration as complementary antimicrobial agents [21]. However, the emergence of multi-drug-resistant bacteria is important to treat microbial infections, so the need to find new substances with antimicrobial properties serves as a novel basis against the microbes [22]. Recent analyses have urged the use of EOs for the therapy of several human health complications due to their promising potential to treat antibiotic resistance and other microbes and as they serve as antidiabetic agents by maintaining human metabolic actions as well as preventing humans from suffering from the many side effects caused due to the intake of chemically synthetic doses [10]. As the plant is medicinally important for local people, therefore, in the current study, we extracted EOs from the stem, flowers, and leaves of the *O. arabicus* and analyzed their volatile chemical constituents. To know the therapeutic potential of this plant, the EOs were further screened for α-glucosidase and antimicrobial activities. The oil of the stem was found more active against the α-glucosidase enzyme, followed by leaves and flowers. To the best of the authors’ knowledge, this is the first report on the chemical composition, α-glucosidase, and antimicrobial activities of this essential oil.

## 2. Materials and Methods

### 2.1. Chemicals and Instrumentation

We obtained the α-Glucosidase enzyme (EC 3.2.1.20, Sigma-Aldrich, Darmstadt, Germany); HEPE Strisbuffer, 4-nitrophenyl acetate, dimethyl sulfoxide (DMSO), sodium sulphate (Na_2_SO_4_), acetazolamide, phosphate-buffered saline, *p*-nitrophenyl-α-d-glucopyranoside, sodium nitroprusside, sodium hydroxide, and sodium hypochlorite (Merck, Darmstadt, Germany); nutrient agar and potato dextrose agar (Sigma-Aldrich Chemie GmbH, Taufkirchen, Germany); and gas chromatography-mass spectrometer (GC-MS-QP2010, Shimadzu, Kyoto, Japan).

### 2.2. Plant Collection and Identification

Various field visits were arranged to the Jabal Al-Akhdar (Ad-Dakhiliyah, 23°6′12.0780″ N, 57°22′47.7984″ E) region in the Sultanate of Oman to harvest the whole plant of *O. arabicus* (8.5 kg) from mid-March to April. The selected plant sample was identified by the taxonomist Dr. Syed Abdullah from the Gilani Department of Biological and Chemical Sciences, University of Nizwa, Oman, using the available literature, and the preserved specimen was deposited at the herbarium (OA/03/2021) of Natural and Medical Science Research Centre at the University of Nizwa Sultanate Oman. 

The selected plant was divided into stem, leaves, and flowers, and was cleaned with tap water to remove undesirable particles and placed in the shade at room temperature for drying. The dried parts were then blended into a fine powder using an electric blender. The obtained powder from various parts of the selected plant was weighed and yielded stem (1200 g), leaves (700 g), and flowers (400 g); they were packed in plastic bags and placed in the refrigerator at 4 °C until further use. 

### 2.3. Essential Oils

The essential oils (EOs) from the stem, leaves, and flowers were extracted via hydro-distillation (three times) employing an 8-quart stove still home distillation unit and Clevenger-type device. The Clevenger machine was operated until no further oil could be extracted. The EOs were collected from the top of the hydrosol and the oils collected in the burette were calculated. Moreover, the obtained oils were passed through an anhydrous sodium sulphate to remove any moisture and placed in the refrigerator to avoid the loss of the essential constituents in the essential oils.

### 2.4. GC-MS Chemical Analysis 

The EO composition from the various parts (stem, leaves, and flowers) of the plant was profiled via GC-MS analysis. The GC-MS device contains the Perkin Elmer Clarus (PEC) 600 GC system, coupled by Rtx-5MS with the capillary column (30 m × 0.25 mm I.D × 0.25 µm film thickness; a maximum temperature of 260 °C), attached to a PEC 600 MS. Ultra-high purity helium (99.99%) was consumed as a carrier gas with a flow rate of 1.0 mL/min. The temperatures of the injection, transfer line, and ion source were 260, 270, and 280 °C, correspondingly. The ionization energy was observed at 70 eV and the electron multiplier (EM) voltage was attained from auto-tune. All the data of the EOs were achieved by screening the full mass spectra within the 45–550 a.m.u. scan range. The volume of the injected sample was 1 µL, having a split ratio of 10:1. The oven temperature was operated at 60 °C (for 1 min) at a rate of 4 °C/min-260 °C held for 4 min. The sample completed its run in 50 min. 

### 2.5. Compound Identification 

The chemical constituents in the EOs of all parts of the selected plant *O. arabicus* were determined through GC-MS and the unknown constituents were identified using mass spectral library software (NIST 2011 v.2.3, Gaithersburg, MD, USA) and with the already reported literature. Moreover, the use of RI (obsd.) compared with RI (Lit.) and co-injection with an available authentic sample of detected compounds to GC or GC/MS were also used for the identification of chemical constituents. The quantification was performed by using an external standard technique via calibration curves produced by operating the GC profiling of the representative compounds.

### 2.6. Antimicrobial Activity 

The significance of the EOs in the *O. arabicus* stem, leaves, and flowers were comparatively examined via disc diffusion using a systematic approach [23]. The cultures for the three fungal and two bacterial strains were refreshed through potato dextrose agar (PDA) and nutrient agar (NA), respectively. Petri plates were covered with a parafilm to avoid contamination, and the fungal (28 °C) and bacterial (37 °C) cultures were checked after 72 and 24 h to screen the antimicrobial bioassay for the tested samples. The inoculum was prepared by the addition of a small colony obtained from the fungal and bacterial strains with normal saline water (0.9% NaCl) in a 1 mL Eppendorf tube and vortexed to make it homogenous. Moreover, the turbidity of the Eppendorf tube was compared with McFarland standard solution. Then, a sterile cotton swab was dipped into the normal saline suspension and well streaked over the entire surface of the plate containing PDA and NA medium to ensure the uniform distribution of the inoculum. Afterwards, the bacterial and fungus inoculation was performed on the plates.

#### 2.6.1. Microbial Strains 

The bacterial strains (ATCC, *E. coli*, and *S. aureus*) and clinically isolated fungal strains (*P. simplicissimum*, *R. solani*, and *F. fujikuroi*) were used in the current study. Streptomycin and Topsin-M 70 WP were applied as the positive control and dimethylsulfoxide (DMSO) was used as the negative control in antimicrobial activity.

#### 2.6.2. Lawn Preparation 

The fungal and bacterial lawn was achieved using PDA and NA media, respectively, and sterilized cotton swabs were used. The EOs from each sample (stem, leaves, and flower) at concentrations of 25, 50, and 100 µL per well were obtained using the disc diffusion method [24]. Furthermore, the bacterial Petri plates were placed at 37 °C and the fungal strains plates were kept at 25 °C in incubators for 24–48 h and inhibition zones (mm) were measured. Streptomycin was used as the positive control in antibacterial activity and Topsin-M 70 WP was used in antifungal activity. DMSO was used as the negative control using the same concentrations.

#### 2.6.3. α-Glucosidase Activity 

The α-Glucosidase activity was performed using 0.1 M phosphate buffer having pH 6.8 at 37 °C [25,26]. The α-glucosidase enzyme (0.2 μg/mL) was incubated in phosphate-buffered saline with various concentrations of EOs at 37 °C for 15 min followed by the addition of 0.7 mM substrate (*p*-nitrophenyl-α-d-glucopyranoside) and the alteration in absorbance was observed for 30 min at 400 nm using a spectrophotometer (Spectra Max M2, Molecular Devices, San Jose, CA, USA). The tested oils were replaced with deuterated dimethylsulfoxide (DMSO-*d*_6_, 7.5% final) in the control. Acarbose was used as the standard (IC_50_ 377.7 ± 1.34 µg/mL). The following formula (1) was used for % inhibition.

% Inhibition = 100 − (OD_test well_/OD_control_) × 100 (1)

### 2.7. Antioxidant Evaluation 

The antioxidant significance of the EOs of the studied samples was proceeded using the 2,2-diphenyl-1-picrylhydrazyl (DPPH) and (ABTS) 2,2-azino-bis (3-ethylbenzothiazoline-6-sulfonic acid assays [1,2,3]. To proceed with the DPPH assay, around 3 mg of DPPH was standardized in 100 mL distilled methanol to form the free radicals and the mixture was placed in the dark for 30 min. The tested samples at dosages of 1000, 500, 250, 125, and 62.5 µg/mL were well-prepared. Next, about 2 mL from the tested samples were added up to 2 mL of the already prepared DPPH stock solution and then again incubated for approximately 20 min in the dark. The absorbance of the tested samples was examined at 517 nm applying UV/Vis spectrophotometer and the % potential was determined via Equation (2).

% Antioxidant activity = 1 − 2/1 × 100(2)

where 1 signifies the control absorbance and 2 denotes the standard absorbance. The ABTS assay was carried out by taking around 383 mg of ABTS and about 66.2 mg of the K_2_S_2_O_8_ separately in 100 mL of MeOH and properly homogenized and, after that, both were combined. Later, around 2 mL of the mixture were arranged to incubate with around 2 mL of examined samples at dosages of 1000, 500, 250, 125, and 62.5 µg/mL for 25 min. Lastly, the absorbance of the analyzed samples was calculated at 746 nm using a UV spectrophotometer, and the significance was estimated using the same Equation (2).

### 2.8. Statistical Analysis

The following programs were utilized to analyze the attained results for biological activity. SoftMax Pro package and Excel were utilized. EZ-FIT (Perrella Scientific, Inc., Amherst, NH, USA) was used for the IC_50_ calculations of all the tested samples. To overcome the expected errors, all experiments were performed in triplicate, and variations in the results are reported as standard error of mean values (SEM), as reported by Akhter et al. [27].

## 3. Results and Discussion

Medicinal plants and their products have promising therapeutic capabilities and serve as a local remedy as well as providing valuable innovative constituents for the mass production of medicines [26,28]. Plant constituents are different since the diverse range of ecological amplitudes alters the composition of the chemical components, which are the promising sources to treat various human health complications [29,30,31].

### 3.1. Essential Oil Composition 

**Figure 1:**
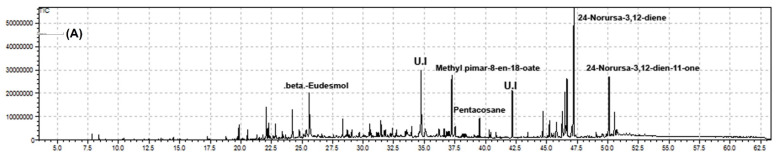

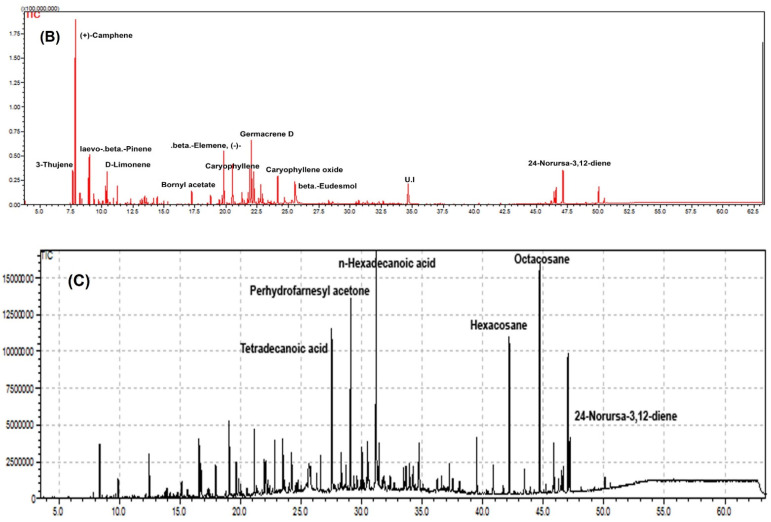
GC-MS chromatogram of *O. arabicus* essential oils: (**A**) flowers, (**B**) stems, and (**C**) leaves.

The EOs obtained from the various parts (flowers, stems, and leaves) of *O. arabicus* had a mild scent and had the appearance of a yellow fluid lighter than water. The GC-MS analysis revealed that the stem, leaves, and flowers of the selected plant are a prominent source of bioactive chemical components. However, a maximum of 74 bioactive compounds were noticed in flowers, contributing 81.46% of the total and whose dominant constituents were 24-norursa-3,12-diene (13.06%), 24-norursa-3,12-dien-11-one (6.61%), and 24-noroleana-3,12-diene (6.25%), followed by the stems with 62 compounds producing 89.52%, with the main constituents being (+)-camphene (21.50%), eremophilene (5.87%), and δ-selinene (5.03%). The least number of compounds were detected in leaves (46), which yields 98.75% of the total oil, with the key compounds being *n*-hexadecanoic acid (12.32%), followed by octacosane (8.62%) and tetradecanoic acid (8.54%). Chromatograms are shown in Figure 1. The chemical components 24-norursa-3,12-diene, 24-norursa-3,12-dien-11-one, 24-noroleana-3,12-diene, methyl pimar-8-en-18-oate, and 24-norursa-3,9(11),12-triene) are reported, up to our best knowledge, for the first time from the flowers essential oil of *O. arabicus*, while having been earlier documented in the literature for the genus Boswellia [18,19,20]. 

**Figure 2:**
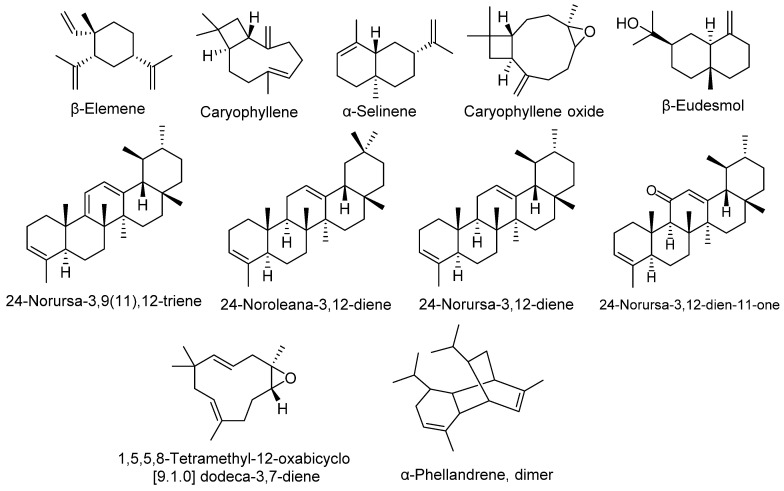
Common compounds identified in all parts of the *O. arabicus*.

The aforementioned bioactive components have significant potential to inhibit the microbial resistance to overcome microbial infections, neutralize the free radicals to prevent cellular damage, and also have promising capacities to act as an anti-cancerous agent, as shown by Hussain et al. [32] and Sonigra et al. [33]. The *O. arabicus* stem contains compounds such as (+)-camphene, eremophilene, δ-selinene, β-elemene, and β-eudesmol, already reported by Chandra et al. [34] in *Callicarpa macrophylla* and Utegenova et al. [35] in some species of the genus *Ferula*, who also highlight their potential against human pathogenic microbes. The EOs of the *O. arabicus* leaves are characterized by *n*-hexadecanoic acid, which was reported earlier by Ravi et al. [36], and is well known for its therapeutic significance. The same parts of *O. arabicus* also contained octaconsane, tetradecanoic acid, perhydro farnesyl acetone, and hexacosane, which have been previously reported by Swamy et al. [37] in *Plectranthus amboinicus* leaves, and have an antimicrobial effect. Moreover, in the present study, it was revealed that all parts (flowers, stems, and leaves) have eleven common compounds, which accounted for 41.85%, 25.82%, and 12.96%, respectively, indicating that these are the prominent compounds present in the plant (Table 1). Some of bioactive compounds showed similarity between the flowers’, stems’, and leaves’ oil constituents (Figure 2). Among these constituents, (+)-camphene with 21.50% is the most dominant compound in the stem of *O. arabicus* and has significant potential against various health complications, which was reported by Vallianou et al. [38], as well as, especially, regulating metabolic disorder, as shown by Noma et al. [39].

### 3.2. Antimicrobial Capabilities

To discover a novel antimicrobial agent, we used EOs of *O. arabicus* stems, leaves, and flowers against the human pathogenic bacterial strains *Escherichia coli* and *Streptococcus aureus*, and the fungal strains *Penicillium simplicissimum*, *Rhizoctonia solani*, and *Fusarium fujikuroi*. As different parts of the same plant have different bioactive compounds, some EOs were active as compared to others (Table 2). Among the tested samples, the EOs of the *O. arabicus* stem showed a significant capacity against the Gram-negative bacteria *E. coli*. This activity was due to the presence of bioactive compounds such as α-campholenal, L-pinocarveol, and myrtenol, which have been earlier reported to have antibacterial potential (Zhang et al. [40], Bansal et al. [41], and Cordeiro et al. [42]). The essential oil of *O. arabicus* leaves also showed better antifungal activity against *P. simplicissimum.* Our results are supported by Sousa et al. [43] as the dominant compound spathulenol in the EOs of *Eugenia calycina* leaf exhibited antibacterial capabilities against the anaerobic Gram-negative bacteria *prevotella nigrescens* in a concentration of 100 µg/mL. Moreover, the antifungal activity of the EOs of the leaves of *O. arabicus* against *P. simplicissimum* and *R. solani* is in complete agreement with the documented results of Yu et al. [44] and Golus et al. [45]. The plant contains caryophyllene, β-caryophyllene, and dodecanoic acid, which have antifungal significance, as reported by Ghaffari et al. [46], Dahham et al. [47], and Wu et al. [48].

### 3.3. In Vitro Antidiabetic Potential

**Figure 3:**
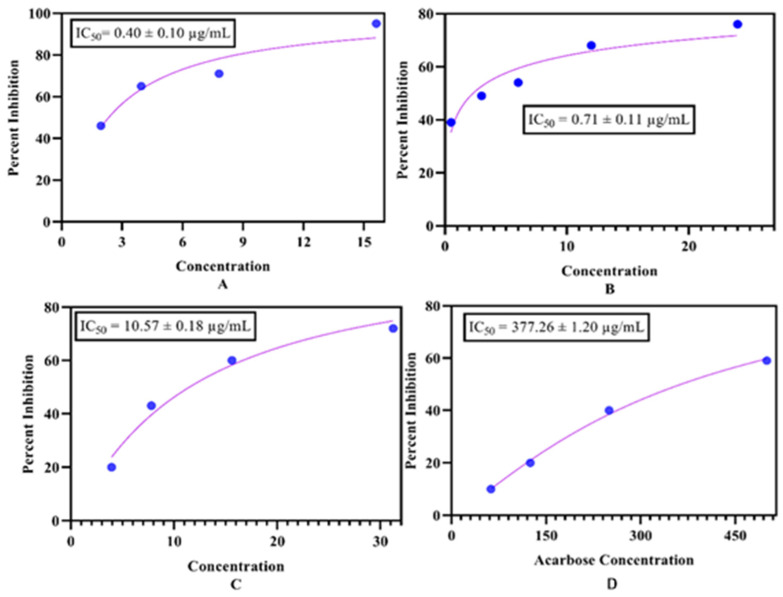
α-Glucosidase significance of the *Ochradinus arabicus* essential oil of (**A**) stems, (**B**) leaves, (**C**) flowers, and the (**D**) standard.

Medicinal plants are rich sources of chemical components that have promising potential to serve as a remedy for various human health complications, including diabetes mellitus. Hence, due to a lack of scientific literature, the EOs of *O. arabicus* stems, leaves, and flowers were investigated for their antidiabetic ability against α-glucosidase (Figure 3). As is known, different parts of plants have variations in the constituents, which is why the EOs extracted from the stem displayed the most potent inhibitory activity (IC_50_ = 0.40 ± 0.10 µg/mL) in comparison with the leaves’ EOs with an IC_50_ value of 0.71 ± 0.11 µg/mL and the EOs of the flowers of *O. arabicus*, with a value of IC_50_ = 10.57 ± 0.18 µg/mL, compared to standard (IC_50_ = 377.26 ± 1.20 µg/mL). Therefore, our findings are the same as those described by Tahir et al. [49] and Akolade et al. [50]. The outcomes of our project also are in accordance with the results of Numonov et al. [51]. The antidiabetic potential is due to the presence of common compounds present in the plant, such as cembrene A, caryophyllene oxide, linalool, nonanal, and dibutyl phthalate, which has been reported before by Marshall et al. [52], Kaur et al. [53] More et al. [54], Chhikara et al. [55], and Keerthana et al. [56], respectively.

### 3.4. Antioxidant Significance

**Figure 4:**
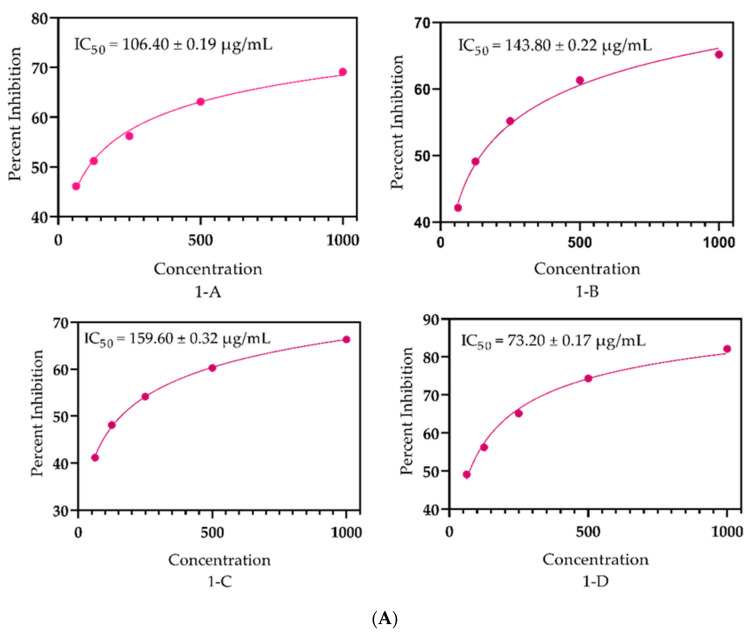

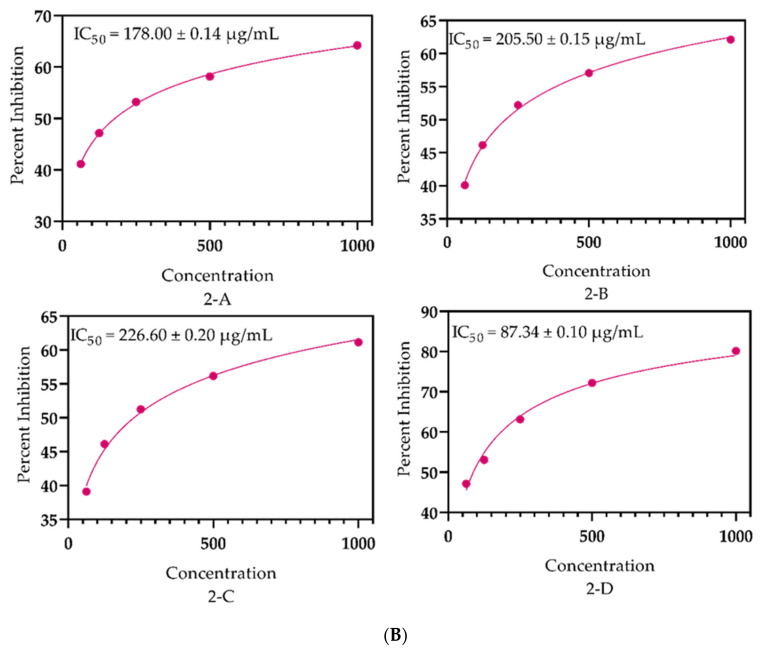
(**A**) Antioxidant potential of the essential oils of *O. arabicus* (**1**-**A**) flowers, (**1**-**B**) leaves (**1**-**C**), stems, and (**1**-**D**) ascorbic acid via the DPPH assay. (**B**) Antioxidant potential of the essential oils of *O. arabicus* (**2**-**A**) flowers, (**2**-**B**) leaves, (**2**-**C**) stems, and (**2**-**D**) ascorbic acid via the ABTS assay.

The essential oils of the studied *O. arabicus* samples (flowers, leaves, and stems) had the free-radical scavenging capabilities, which were assessed using DPPH and ABTS bioassay. The EOs had considerable potential in both assays (Figure 4A,B). The flowers of the plant under study presented the highest significance with IC_50_ = 106.40 ± 0.19 µg/mL, followed by the leaves and stems with an IC_50_ = 143.80 ± 0.22 µg/mL and 159.60 ± 0.32 µg/mL, respectively, as compared with the standard, with an IC_50_ = 73.20 ± 0.17 µg/mL. Moreover, in the ABTS assay, the flowers of the selected plant had, followed by the leaves and stems, an IC_50_ = 178.0 ± 0.14, 205.50 ± 0.15, and 226.60 ± 0.20 µg/mL, respectively. Furthermore, the utmost effect was detected in the DPPH assay (Figure 4A) with IC_50_ = 73.20 ± 0.17 µg/mL in comparison with the ABTS assay IC_50_ = 87.34 ± 0.10 µg/mL (Figure 4B). The capacity to scavenge free radicals is mainly attributed due to the presence of α-pinene, (+)-camphene, and thymol, which have promising capacities to neutralize free radicals as stated by Wang et al. [4], Yang et al. [5], and Yildiz et al. [6], respectively. The plant extract has already been reported for its antioxidant impact, as stated by Alshamsi et al. [7], but the essential oils composition through GC-MS analysis of the selected plant parts were applied here for the first time. Our findings are in agreement with the data of Ben Nouri et al. [8] for *Cupressus sempervirens* and of *Nigella sativa* as reported before by [9]; other essential oils extracted from other plants were described by Anthony et al. [10]. However, our outcomes have little variation from the literature, especially that presented by Thusoo et al. [11] for *Valeriana jatamansi* and Diniz do Nascimento et al. [12] for some spice plants, which were studied for their free-radical scavenging potential. The chemical components in a plant are responsible for addressing various ailments, including antioxidant potential [13,14], whose contents are influenced by various factors such as edaphic, climatic, and topographic factors, as shown by Sampaio et al. [15]. The quantity of the active ingredients may be affected due to the quality of water, as shown in the work of Ghani et al. [16].

## 4. Conclusions

EOs contain bioactive constituents that serve as a basis for the pharmaceutical trade, nutraceutical supplements, and due to their fragrance, are required for producing cosmetics and perfumes. They also have a significant role in resisting microbes as well as regulating metabolic disorders and scavenging free radicals, acting as a natural remedy. The EOs of *O. arabicus* presented significant bioactive compounds in all parts (stems, leaves, and flowers). Among the tested samples, the *O. arabicus* flowers’ EOs had seventy-four chemical constituents, ensued by the stem EOs with sixty-one compounds, while a minimum of fifty-one compounds were found in the leaves’ EOs. In conclusion, (+)-camphene (21.50%) was the major compound detected in the stems’ EOs, hexadecanoic acid (12.32%) in the EOs of leaves, and 24-Norursa-3,12-diene (13.06%) in the flowers’ oils. The stems’ EOs of *O. arabicus* were found effective against the human pathogenic bacterial strain *E. coli* and the fungal strain *R. solani*. In addition, the other tested samples were observed to be inactive against the examined microbes, while substantial in vitro α-glucosidase activity was noticed in all parts of the plant and, particularly, the EOs of the *O. arabicus* stems can act out as an antidiabetic agent. The essential oils have a promising potential to scavenge free radicals. However, additional studies are still essential to emphasize and isolate new chemical constituents responsible for the observed activities.

## Figures and Tables

**Table 1:** The GC-MS-based identified compounds in flowers (F), stem (S), and leaves (L) of *O. arabicus*.

Name of the Compound	^a^ RI_cal._	^b^ RI_rep._	F (%)	S (%)	L (%)
5,5-Dimethyl-1-vinylbicyclo[2.1.1]hexane	922	920	-	0.09	-
Cyclene	925	922	-	0.02	-
3-Thujene	930	928	-	2.65	-
α-Pinene	932	931	0.38	-	-
(+)-Camphene	936	935	-	21.50	-
2,4(10)-Thujadiene	962	957	-	0.43	-
Butyl isothiocyanate	962	959	0.36	-	-
Sabinene	966	964	-	1.92	-
2,4-heptanedienal	968	966	-	-	0.61
β-Pinene	972	970	0.02	4.36	-
Isobutyl isothiocyanate	975	974	-	-	1.64
β-Myrcene	980	979	0.03	0.82	-
α-Phellandrene	1002	997	0.03	0.43	-
3-Carene	1008	1005	-	0.09	-
*p*-Cymene	1014	1011	-	1.32	-
(+)-4-Carene	1020	1018	0.03	-	-
o-Cymol	1022	1025	0.08	-	-
d-Limonene	1018	1020	0.14	2.54	-
γ-Terpinene	1048	1047	0.05	1.32	-
α-Dimethylstyrene	1072	1070	0.07		-
Linalool	1080	1081	0.02	0.41	-
*n*-Nonanal	1082	1082	0.08		-
Perillen	1087	1086	-	0.04	-
α-Campholenal	1100	1102	0.04	0.36	-
2,9-Dimethyl-5-decyne	1102	1103	-	0.43	-
l-Pinocarveol	1105	1106	0.17	0.66	-
*cis*-Verbenol	1108	1110	-	0.18	-
Nonanal	1120	1128	-	-	1.50
Trans-verbenol	1130	1132	0.06	0.53	-
2-nonenal	1132	1133	-		0.30
Terpineol	1142	1143	0.06		-
α-Phellandren-8-ol	1148	1148	0.04	0.45	-
Terpinen-4-ol	1174	1175	0.18	0.58	-
α-Terpineol	1171	1172	0.06	-	-
Myrtenol	1176	1175	0.06	0.34	-
Decanal	1182	1183	-	-	0.50
2,6,6-trimethyl-1-cyclohexene-1-carboxaldehyde	1190	1196	-	-	0.46
Levoverbenone	1200	1208	-	0.22	
Decanal	1238	1240	-	-	1.96
Nonanoic acid	1266	1268	-	-	1.93
*cis*-Carveol	1268	1269	-	0.02	-
Bornyl acetate	1270	1270	0.28	1.09	-
Thymol	1264	1266	0.09	-	-
2,4-decadienal	1290	1291	-	-	1.19
α-Terpinyl acetate	1320	1322	0.25	0.81	
2-undecenal	1335	1337	-	-	3.07
Copaene	1375	1376	0.42	-	-
β-Bourbonene	1380	1386	0.21	0.81	-
6,10-dimethyl-2-undecanone	1388	1389	-	-	0.77
β-Elemene	1399	1398	1.31	5.08	1.11
Caryophyllene	1420	1421	0.72	3.97	0.41
β-Caryophyllene	1422	1423	-	-	0.30
*cis*-Geranylacetone	1426	1427	-	-	2.14
Humulene	1455	1454	0.41	1.18	-
Alloaromadendrene	1458	1459	0.18	0.38	-
trans-β-Ionone	1460	1462			1.14
γ-Muurolene	1470	1471	0.29	0.92	
β-Selinene	1477	1478	-		1.20
Germacrene D	1480	1480	-	3.30	-
β-Eudesmene	1480	1483	2.51	-	-
Eremophilene	1485	1486	-	5.87	-
Pentadecane	1500	1500	0.66	-	-
α-Selinene	1502	1502	1.23	2.97	0.41
Pentadecane	1504	1504	-	-	1.14
γ-Cadinene	1506	1507	0.31	-	-
δ-Selinene	1508	1509	-	5.03	-
Cubebol	1510	1512		0.41	0.32
δ-Cadinene	11,512	1514	1.12	1.52	-
Elemol	1534	1535	0.65	0.44	-
Dodecanoic acid	1552	1554	-	-	2.50
Ledol	1560	1561	0.28	-	
Carotol	1566	1568	-	-	2.01
Germacrene D-4-ol	1570	1570	-	0.06	
Caryophyllene oxide	1574	1575	2.29	2.41	1.48
Viridiflorol	1595	1594	-	0.52	
Hexadecane	1600	1600	-		1.02
1,5,5,8-Tetramethyl-12-oxabicyclo [9.1.0] dodeca-3,7-diene	1601	1601	0.63	0.31	0.43
Epicubenol	1620	1621	0.24		-
γ-Eudesmol	1626	1627	0.55	0.31	-
(-)-Cubenol	1630	1631	0.49	0.13	-
tau-Cadinol	1636	1637	1.22	0.39	-
α-Eudesmol	1642	1643	2.71		-
β-Eudesmol	1643	1644	4.02	4.79	3.02
δ-Cadinol	1645	1646	0.27	2.21	
β-Cyperone	1705	1706	0.23	-	-
Tetradecanal	1760	1760	-	-	1.20
Benzyl Benzoate	1764	1765	-	0.09	
Tetradecanoic acid	1770	1772	-		8.54
α-Phellandrene dimer	1800	1801	1.45	0.33	1.33
Perhydro farnesyl acetone	1840	1842		-	7.27
octadecamethylcyclononasiloxane	1865	1865	0.66	-	-
Thunbergen	1920	1924	0.53	-	-
*n*-Hexadecanoic acid	1942	1940	-	-	12.32
Linalyl phenylacetate	1942	1945	1.04	-	-
*m*-Camphorene	1960	1960	-	0.21	-
Cembrene A	1970	1968	-	0.28	-
Verticilla-4(20),7,11-triene	1885	1985	-		0.54
*p*-Camphorene	1992	1994	0.35	0.14	
Methyl palmitate	1908	1909	1.09	-	0.930
α-Kaurene	2005	2006	0.47	-	-
α-Pinacene	2018	2019	0.35	-	-
α-Springene	2012	2013	0.36	-	-
Eicosamethylcyclodecasiloxane	2024	2025	1.82	-	-
Isocembrol	2070	2071	0.59	-	-
Thunbergol	2072	2073	-	-	0.33
2,13-Octadecadien-1-ol	2074	2074	-	-	1.70
Heneicosane	2081	2084	-	-	0.85
2-Nonadecanone	2086	2087	-	-	0.43
γ-Palmitolactone	2093	2100	-	-	1.14
Verticiol	2105	2106	0.88	0.26	-
Methyl-7,10-octadecadienoate	2100	2101	0.41	-	-
Heneicosane	2104	2102	-	-	1.38
Cembrenol	2160	2161	-	0.07	-
Linolenic acid, 2-hydroxy-1-(hydroxymethyl)ethyl ester	2162	2162	0.57	-	-
Ethyl linoleate	2165	2166	0.57	-	-
Methyl pimar-8-en-18-oate	2230	2231	5.88	-	-
Tricosane	2275	2300	-	-	0.53
3,7,11,15-Tetramethyl-2E,6E,10E,14-hexadecatetraenyl acetate	2300	2301	-	-	1.76
Tetracosane	2380	2400	-	-	1.92
Cembra-2,7,11-trien-4,5-diol	2424	2428	-	-	0.61
Nonacosane	2900	2900	1.57	-	-
Diisooctyl phthalate	2524	2525	0.73	-	-
1-Hexacosene	2594	2596	-	-	1.14
Hexacosane	2600	2600	-	-	6.00
2-Methylhexacosane	2662	2664	0.41	-	-
Tetracosane	2702	2700	-	-	1.00
Octacosane	2804	2800	-	-	8.62
Epiandrosterone	2896	2897	1.69	-	-
Nonacosane	2900	2900	-	-	1.88
3-(3,4-Dimethylphenyl)-3-methylandrostan-17-one	2910	2912	0.29	-	-
Cycloart-23-ene-3,25-diol	3070	3071	1.79	-	-
24-Norursa-3,9(11),12-triene	3042	3042	4.28	1.23	0.90
24-Noroleana-3,12-diene	3056	3057	6.25	1.52	1.18
24-Norursa-3,12-diene	3100	3105	13.06	4.26	2.21
24-Norursa-3,12-dien-11-one	3300	3351	6.61	1.94	0.48
β-Amyrone	3370	3372	2.23	0.61	
Total oil components (%)			81.46	95.95	98.75

RI_(calc)_ = Retention index (calculated); ^a^ Elution order on Rtx-5MS capillary column; ^b^ RI = Retention index obtained from the database (NIST, 2011).

**Table 2:** Antimicrobial significance of the EOs of *O. arabicus*.

**Antibacterial Significance**				
Sample used	*E. coli*		*S. aureus*	
	Mean ± SD (mm)			
	50 µL	100 µL	50 µL	100 µL
Stem	15 ± 0.15	19.7 ± 0.12	-	-
Leaves	-	-	-	-
Flowers	-	-	-	-
Streptomycin	23 ± 0.13	30 ± 0.26	22 ± 0.31	29 ± 0.04
DMSO	-	-	-	-
**Antifungal Potential**				
Sample used	*R. solani*		*P. simplicissimum*	
	50 µL	100 µL	50 µL	100 µL
Stem	15 ± 0.14	19.7 ± 0.15	-	-
Leaves	-	-	-	-
Flowers	-	-	-	-
Topsin-M 70 WP	23 ± 0.11	30 ± 0.22	22 ± 0.18	29 ± 0.08
DMSO	-	-	-	-

EOs = essential oils; SD = standard deviation; DMSO = dimethyl sulfoxide; positive controls: streptomycin for antibacterial and Topsin-M 70 WP for antifungal activities.

## Data Availability

The data presented in this study are available in the article.

## Sample Availability

Samples of the compounds are not available from the authors.
